# Efficient loading of dendritic cells following cryo and radiofrequency ablation in combination with immune modulation induces anti-tumour immunity

**DOI:** 10.1038/sj.bjc.6603341

**Published:** 2006-09-05

**Authors:** M H M G M den Brok, R P M Sutmuller, S Nierkens, E J Bennink, C Frielink, L W J Toonen, O C Boerman, C G Figdor, T J M Ruers, G J Adema

**Affiliations:** 1 Department of Tumor Immunology, Nijmegen Centre for Molecular Life Sciences, Radboud University Nijmegen Medical Centre, PO Box 9101, Nijmegen 6500 HB, The Netherlands; 2 Department of Surgery, Radboud University Nijmegen Medical Centre, PO Box 9101, Nijmegen 6500 HB, The Netherlands; 3 Department of Nuclear Medicine, Radboud University Nijmegen Medical Centre, PO Box 9101, Nijmegen 6500 HB, The Netherlands

**Keywords:** vaccination, dendritic cell, CTLA-4, regulatory T cell, radiofrequency ablation, cryo ablation

## Abstract

Dendritic cells (DC) are professional antigen-presenting cells that play a pivotal role in the induction of immunity. *Ex vivo*-generated, tumour antigen-loaded mature DC are currently exploited as cancer vaccines in clinical studies. However, antigen loading and maturation of DC directly *in vivo* would greatly facilitate the application of DC-based vaccines. We formerly showed in murine models that radiofrequency-mediated tumour destruction can provide an antigen source for the *in vivo* induction of anti-tumour immunity, and we explored the role of DC herein. In this paper we evaluate radiofrequency and cryo ablation for their ability to provide an antigen source for DC and compare this with an *ex vivo*-loaded DC vaccine. The data obtained with model antigens demonstrate that upon tumour destruction by radiofrequency ablation, up to 7% of the total draining lymph node (LN) DC contained antigen, whereas only few DC from the conventional vaccine reached the LN. Interestingly, following cryo ablation the amount of antigen-loaded DC is almost doubled. Analysis of surface markers revealed that both destruction methods were able to induce DC maturation. Finally, we show that *in situ* tumour ablation can be efficiently combined with immune modulation by anti-CTLA-4 antibodies or regulatory T-cell depletion. These combination treatments protected mice from the outgrowth of tumour challenges, and led to *in vivo* enhancement of tumour-specific T-cell numbers, which produced more IFN-*γ* upon activation. Therefore, *in situ* tumour destruction in combination with immune modulation creates a unique, ‘*in situ* DC-vaccine’ that is readily applicable in the clinic without prior knowledge of tumour antigens.

Dendritic cells (DC) are crucial antigen-presenting cells (APC) for the initiation of primary T-cell responses (Adams *et al*, 2005; Banchereau and Palucka, 2005). Immature DC are well equipped to take up and process antigen from their surroundings, but they lack sufficient co-stimulatory signals required for productive T-cell activation. In a stimulatory environment, like in an infection, immature DC undergo activation, maturation and acquire the capacity to cross-present exogenous antigens in MHC class I. Particularly, the Toll-like receptor (TLR) family of proteins initiates the DC maturation process upon recognition of conserved pathogen-associated molecular patterns (PAMPs), like LPS or unmethylated CpG oligodeoxynucleotides. Upon maturation, co-stimulatory molecule and MHC-peptide complex expression increases and cytokines like IL-12 skew the functional outcome of the response (Trinchieri, 2003). Dendritic cells that did not perceive an activating environment while taking up antigen do not mature and induce tolerance rather than immunity. The importance of immune activation for the induction of anti-tumour immunity has been well established. Next to direct activation of DC by PAMPs, a broad range of indirect strategies has been explored to accomplish activation of the immune system. Expression of, for instance, co-stimulatory molecules on tumours can induce T-cell-mediated rejection of a variety of tumours (Townsend and Allison, 1993). In addition to these stimulatory pathways, also blockade of inhibitory receptors, for example, CTLA-4, has been applied successfully to induce tumour rejection (Chen *et al*, 1992; Egen *et al*, 2002). In this respect, also *in vivo* depletion of regulatory T cells, that are able to suppress conventional T-cell expansion, has been shown to evoke anti-tumour immunity (Sutmuller *et al*, 2001; Sakaguchi, 2005).

As murine models demonstrate that especially DC are effective in inducing effective immune responses, *ex vivo* generated DC are currently applied to stimulate anti-tumour immunity in clinical trials (Banchereau *et al*, 2000; Coulie and van der Bruggen, 2003; De Vries *et al*, 2003; Schuler *et al*, 2003; Figdor *et al*, 2004). Although tumour-specific responses have been obtained with tumour antigen-loaded DC-based vaccines, many questions remain unanswered (Figdor *et al*, 2004; Steinman and Mellman, 2004). Especially the migration of *ex vivo* generated DC-based vaccines from i.d./subcutaneously (s.c.) injected depots to the draining lymph nodes (LN) has been shown to be inefficient in both mouse models and patients (Eggert *et al*, 1999; Josien *et al*, 2000; De Vries *et al*, 2003). Moreover, *ex vivo* generation and loading of DC is time consuming and costly. *In vivo* loading and maturation of DC would therefore improve the applicability of DC vaccination to a great extend.

Recent studies using antigens coupled to antibodies directed against the mouse DC antigen DEC-205 (Steinman and Pope, 2002) or attraction of DC to the tumour via retrovirus-mediated expression of the DC-attracting chemokine CCL20 (Furumoto *et al*, 2004) illustrate the possibility to directly load tumour antigens onto DC *in vivo*. We previously showed in a murine model that the tumour debris left in the body after *in situ* tumour destruction by radiofrequency ablation is an *in vivo* tumour antigen source for the immune system. Excision of the ablated tumour largely prevented the induction of anti-tumour immunity (unpublished observation). Adoptive transfer experiments demonstrated that the immunity induced is T-cell-dependent. Dendritic cells appeared to play an important role in the initiation of this immune response. Tumour ablative treatments, like cryo or radiofrequency ablation, are successfully used in clinical settings to destruct different types of tumours (Zagoria *et al*, 2001; Ruers and Bleichrodt, 2002; Curley, 2003; Garcea *et al*, 2003; Raj *et al*, 2003). Nevertheless, cancer patients treated with ablative regimens mostly develop systemic recurrences as a consequence of the outgrowth of distant micro-metastases, implying that in general no protective immune response is induced. This observation is consistent with our findings that efficient induction of immunity following ablation requires that additional immune activation stimuli are given simultaneously (den Brok *et al*, 2004).

We now show that *in situ* tumour destruction by either cryo or radiofrequency ablation can be employed to efficiently provide antigens to DC *in vivo*. A side-by-side comparison of the two ablative techniques demonstrates that following cryo ablation of established B16 tumours (5–7 mm) up to 13% of DC in the draining LN acquire tracer proteins as a marker of tumour-derived antigen. Radiofrequency ablation results in 7% of LN DC being loaded with the marker antigen. With both ablation methods far more marker antigen-containing DC are detected in the draining LN than with a conventional *in vitro* generated DC vaccine. We further show that both destruction methods in itself were able to enhance DC maturation *in vivo* to an equal extend, comparable to *ex vivo* maturation of DC. Finally, we report that both tumour ablation techniques can be efficiently combined with immuno-modulatory approaches, like blockade of CTLA-4 signalling or regulatory T-cell depletion, to induce functional CD8+ T cells creating systemic anti-tumour-immunity.

Therefore, *in situ* tumour destruction by cryo or radiofrequency ablation combined with immuno-modulatory approaches constitutes a powerful ‘*in situ* DC-vaccine’ for which no prior knowledge of tumour antigens is needed.

## MATERIALS AND METHODS

### Animals

Nine- to 11-week-old female C57BL/6n mice were purchased from Charles River Wiga (Sulzfeld, Germany). Animals were held under specified pathogen-free conditions in the Central Animal Laboratory (Nijmegen, The Netherlands). All experiments were performed according to the guidelines for animal care of the Nijmegen Animal Experiments Committee.

### Tumours

Mice were injected s.c. at the right femur with 500 × 10^3^ cells of the OVA-transfected murine melanoma cell line B16F10 (B16-OVA, clone MO5), which was kindly provided by Dr Kenneth Rock (Falo *et al*, 1995), or wt B16F10. Cells were cultured and injected as described before (den Brok *et al*, 2004). Tumour volumes were scored every 3 days with the formula (AxB^2^) × 0.4, in which A is the largest and B is the shortest dimension. Tumours were selected for ablation when their diameter measured between 5 and 7 mm (d9–10) and only if the tumour was relatively round (>98% of mice).

### Radiofrequency ablation

Animals were anaesthetised by isoflurane inhalation and properly shaved at the tumour area and on the contra-lateral flank. After placement and proper attachment of the contra lateral side onto an electricity-conducting pad (grounding pad), the tumour area was disinfected with alcohol. A radiofrequency ablation needle with active tip of 8 mm (SMK-15, Cotop, Amsterdam, The Netherlands) was inserted s.c. and placed in the middle of the tumour. After placement of the radiofrequency ablation needle, impedance could be evaluated on the radiofrequency lesion generator system (Model RFG-3B, Radionics, Burlington, MA, USA). Next, treatment was started by delivering radiofrequency energy. During a treatment cycle of ±80 s, temperature could be monitored by means of a thermistor and thermocouple in the tip of the probe. Treatment was considered successful if a tip temperature of 75–80°C could be reached.

### Cryo ablation

Animals were properly shaven and anaesthetised by isoflurane inhalation. The tumour area was disinfected with alcohol and subsequently wetted with distilled water. The tip of the liquid nitrogen cryo ablation system (CS76, Frigitronics, Shelton, CT, USA) was placed onto the tumour and after proper freeze attachment, treatment was started. During two treatment cycles of ±70 s the tumour and a small strip around it were frozen to less than −100°C. Treatment was considered successful when the whole tumour appeared frozen macroscopically.

### Re-challenge model

Forty days after ablation of B16-OVA tumours, mice were challenged by s.c. injection at the contra-lateral femur of either 15 × 10^3^ B16-OVA cells or 15 × 10^3^ EL4 cells (numbers defined by titration). Injections were performed in 100 *μ*l PBS. Mice were killed when tumours reached a volume of ±850 mm^3^.

### ^111^Indium conjugation and antigen monitoring

KLH (Calbiochem, Darmstadt, Germany) (10 mg ml^−1^) was conjugated with 240 *μ*g cDTPA (Sigma-Aldrich, Zwijndrecht, The Netherlands) in 0.1 M NaHCO_3_, pH 8.2, during 30 min. Unconjugated DTPA was removed by dialysis against 0.1 M Na-citrate buffer, pH 5.0. The KLH-DTPA conjugate (1.5 mg) was incubated with 1.5 mCi ^111^InCl_3_ (Mallinckrodt, Petten, The Netherlands) in 1.2 ml 0.1 M Na-citrate buffer, pH 5.0, during 30 min. Similar procedures were followed for the ^111^In-OVA conjugate. Radiochemical purity of each preparation (>95%) was determined by instant thin-layer chromatography (ITLC, Gelman Sciences Inc., MI, USA). For antigen monitoring experiments, mice received intratumoural (i.t.) injections of OVA/KLH labelled with ^111^In (^111^In-OVA, ^111^In-KLH) or ovalbumin conjugated to Alexa-488 (OVA-Alexa-488) (Molecular Probes, Leiden, The Netherlands). Conjugates (20 *μ*g (=20 *μ*Ci)) were injected directly before ablation in 20 *μ*l PBS. At various time points after injection of the protein conjugates, mice were anesthetised and scintigraphic images were acquired using a *γ*-camera with ^111^In collimator (Siemens Orbiter, Siemens Inc. Hoffmann Estate, IL, USA) as described previously (Eggert *et al*, 1999). For biodistribution studies mice were killed at different time points after injection of the protein conjugates. Liver, kidney, spleen, draining LN (r. sup. ing.) and non-draining LN (l. sup. ing.) were collected, weighed and counted in a *γ*-counter (1480 Wizard, Wallac Oy, Finland). Injection standards were taken for physical decay correction. In DC sorting experiments, mice received ^111^In-KLH i.t., and at different time points post-ablation CD11c+ DC were sorted as described below and counted in the *γ*-counter.

### Magnetic bead cell sorting and flow cytometric analysis

For antigen uptake experiments (^111^In-KLH and OVA-Alexa), draining LN's from five to eight mice were pooled and after crushing, dissociation in DNAse/collagenase/EDTA, and passage through nylon mesh (Vremec *et al*, 2000), cells were counted and sorted by standard MACS isolation with a MACS Midi column (Miltenyi Biotec). Positive selection of DC was done using CD11c beads (clone N418, Miltenyi Biotec, B.Gladbach, Germany), whereas negative selection/enrichment was done on the CD90 T-cell marker (Thy1.2, 30.H12, Miltenyi Biotec). Sorts were verified by CD3e or CD11c (HL3) staining (not shown). Subsequently, cells were stained and analysed on a FACS-Calibur™ system (BD) with the CELLQuest software. Stainings were performed using the following mAbs: CD11c-APC (HL3), CD8b-FITC (53–5.8), CD3e-PE (17-A2), biotinylated CD80 (1G10) and streptavidin-PE. All antibodies were purchased from BD Pharmingen (Alphen a/d Rijn, The Netherlands).

### Dendritic cell culture from green fluorescent protein transgenic mice

GFP-expressing DC were cultured and injected as described elsewhere (Eggert *et al*, 2003). Briefly, bone marrow was collected from GFP-transgenic mice and cultured for 7 days in the presence of GM-CSF and IL-4. At day 7, 1 *μ*g ml^−1^ LPS was added for 24 h maturation. Next, the non-adherent fraction was harvested, washed and loaded for 1 h with the K^b^-peptide of OVA (SIINFEKL). Cells (1 × 10^6^) were injected s.c. or peri-tumourally (p.t.) at the femur. Isolation of LN cells and sorting was identical as described above.

### Antibody treatments

In the anti-CTLA-4 treatment mice received anti-CTLA-4 antibody (clone 9H10 (Egen *et al*, 2002)) at days 0, 3 and 6 after ablation. Treg depletion was performed by injection of anti-CD25 antibodies (clone PC61, (Onizuka *et al*, 1999)) 4 days before ablation. Injections (200 *μ*g) were done intraperitoneal in PBS. In all cases depletion was successful as verified by FACS (not shown).

### Tetramer staining and interferon-*γ* measurement

A T-cell culture was obtained from spleen and draining LN's of mice 10 days after ablation of a B16-OVA tumour or from naïve control mice. Stimulation of these cells (100 × 10^3^) was performed by addition of irradiated, IFN-*γ*-treated, B16-OVA cells (50 × 10^3^) in IL-2 (10 CU ml^−1^) supplemented culture medium. At days 5 and 10, cells were collected and cleaned in a density gradient. At day 10 of culture, cells were stained for 15 min at RT by OVA-tetramers (H-2K^b^) conjugated to APC (Pelimers, Sanquin, Amsterdam, The Netherlands), counterstained with CD8b, and analysed by FACS. Same bulk cultures were used to collect supernatant 24 h after stimulation with irradiated B16-OVA cells. Capture and biotinylated detection antibodies directed to mouse IFN-*γ* were purchased from BD Pharmingen and, using standard ELISA procedures, IFN-*γ* concentration was measured in 50 *μ*l of supernatant.

### Statistical analysis

Data were analysed for statistical significance by Student's *t* test, except for the Kaplan–Meier survival curves for which a log rank test was used.

## RESULTS

### Immune responses following radiofrequency or cryo ablation

**Figure 1:**
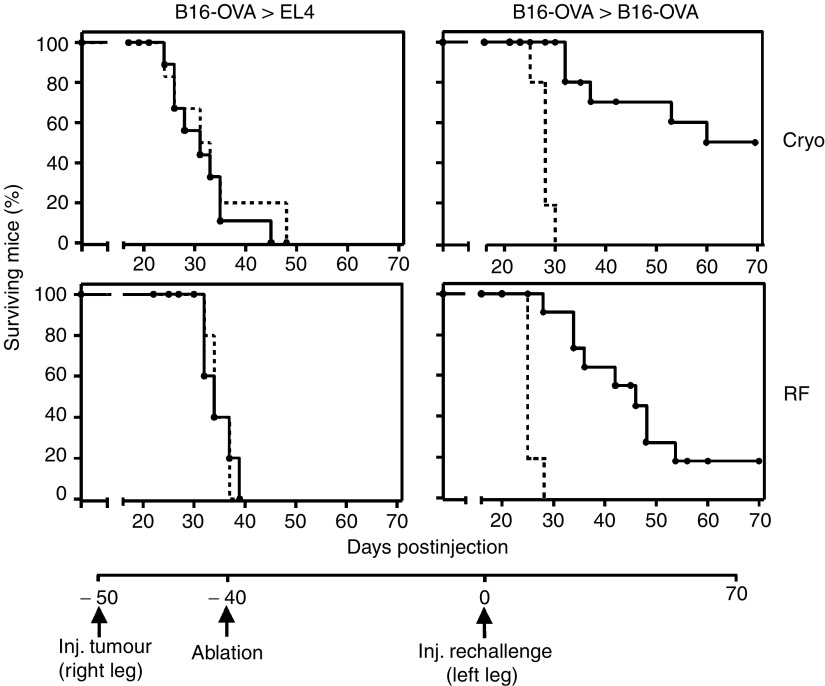
Anti-tumour immunity following radiofrequency or cryo ablation. Mice with established B16-OVA melanomas (5–7 mm) were ablated by cryo (cryo, upper panels) or radiofrequency ablation (RF, lower panels). Forty days later, a re-challenge with 15 × 10^3^ EL4 (left panels) or 15 × 10^3^ B16-OVA cells (right panels) was given s.c. in the contra-lateral leg. Figures depict survival curves demonstrating specificity of the response and growth reduction/protection after ablation. As a control, tumour growth was monitored by injection of the same tumour dose into naïve mice (dotted lines). *T*=0 corresponds to the time of injection of the tumour re-challenge. *P*<0.005 for both B16 lines *vs* control. One out of four representative experiments is shown (*n*=5–11).

We previously demonstrated that radiofrequency ablation of established (5–7 mm) murine tumours resulted in weak, but tumour-specific anti-tumour reactivity. However, the mechanism by which immunity is induced and the role of DC herein remain largely unknown. Therefore, we explored the fate of tumour debris generated by two distinct tumour ablation approaches and the role of DC in the subsequent induction of immune responses. To first compare the induction of immunity after both techniques, B16-OVA tumour-bearing mice were treated with either radiofrequency or cryo ablation and then re-challenged with either B16-OVA cells or non-related EL4 thymoma cells. A detailed time schedule is given below Figure 1. Re-challenges were given 40 days after ablation to exclude direct effects of the ablations on the tumour re-challenge. As shown in Figure 1, radiofrequency ablation of B16-OVA resulted in a clear delay in the outgrowth of B16-OVA tumour cells as compared to naïve controls and a low level of protection (20% of the mice, lower right panel). Interestingly, when mice received cryo ablation, slightly more mice were protected (50% of the mice, upper right panel). In contrast, no delay in outgrowth of the non-related EL4 mouse thymoma was observed (left panels). These data imply that a weak, but tumour-specific immune response had developed after both *in situ* tumour destruction techniques. In all experiments, cryo ablation was slightly more effective than radiofrequency ablation.

### Efficient *in vivo* antigen acquisition by lymph node CD11c(+) dendritic cell following ablation

**Figure 2:**
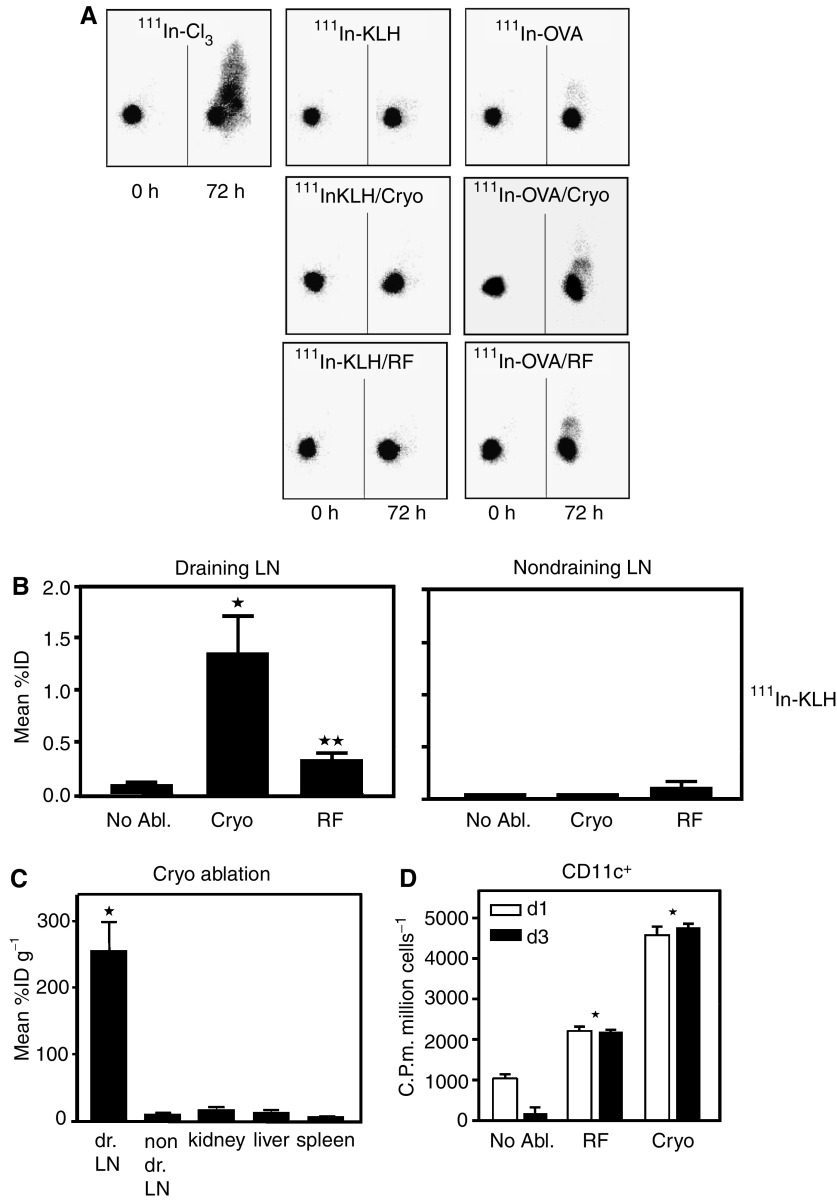
Preferential uptake of tumour-derived antigens by LN DCs. (**A**) To study the fate of tumour antigens after ablation, 20 *μ*Ci of ^111^In-Cl_3_ (left panel), ^111^In-KLH (middle panels) or ^111^In-OVA (right panels) was injected into established B16-OVA tumours (5–7 mm). Tumours were left untreated or ablated by cryo ablation (cryo) or radiofrequency ablation (RF) directly after these injections. *γ*-Camera imaging was performed at the indicated time points. For ^111^In-Cl_3_-injected mice the contours, tumour and liver are visible. One representative mouse out of three is shown. (**B** and **C**) Biodistribution of ^111^In-KLH was determined in dissected LNs and organs of mice injected i.t. 1 day before. Tumours were either left untreated or ablated directly after KLH injection. Radioactivity values from LN's of four mice per group are presented as mean percentages of injected dose with s.d., whereas the values for the organs are also corrected for weight. Mice in panel **C** received cryo ablation, but comparable results were obtained with RF ablation. (**D**) Lymph node suspensions from non-ablated and ablated mice (five mice pooled per group) that received i.t. ^111^In-KLH were subjected to magnetic bead sorting of CD11c(+) cells. After sorting at days 1 and 3 after ablation, the cell-associated radioactivity was measured in the CD11c(+) and CD11c(−) (not shown) cell fraction. Values are presented as counts per minute, corrected for 1 × 10^6^ cells and natural decay, with s.d. from triplicates. ^*^=*P*<0.005 compared to no ablation. In all figures one out of three representative experiments is shown.

To determine the fate of tumour antigens and the involvement of DC in the observed anti-tumour immune responses, we studied *in vivo* antigen acquisition by DC following radiofrequency and cryo ablation. Mice carrying established B16-OVA tumours (5–7 mm) received an i.t. injection of ^111^Indium-labelled KLH or ^111^In-OVA tracer proteins before ablation to monitor the fate of tumour-debris. *γ*-Camera imaging demonstrated that ^111^In proteins remained in the tumour for at least 72 h, whereas unbound ^111^InCl_3_ rapidly distributes throughout the mice (Figure 2A). Even following both ablation procedures ^111^In-KLH and OVA largely remained at the treated site whereas only minor local spreading was observed. In case ^111^In-OVA was used slightly more spreading to the liver was seen compared to KLH, which is likely dependent on the molecular characteristics of OVA. Biodistribution analysis revealed an ablation-dependent accumulation of radioactivity in the draining LN's when ^111^In-KLH (Figure 2B–D) or ^111^In-OVA (not shown) was used. Although significantly different from untreated tumour-bearing mice, radiofrequency-ablated mice showed significantly less accumulation of radioactivity compared to cryo ablation (Figure 2B). Little or no activity was found in the non-draining LN's, liver, kidneys or spleen with or without ablation (Figure 2C), whereas the organs contained high concentrations of radioactivity following injection of unbound ^111^InCl_3_ (not shown).

Applying magnetic bead sorting on the pan-DC marker CD11c we analysed the cells in the draining LN's containing ^111^In-KLH. As shown in Figure 2D, following ablation the cell-associated radioactivity was largely present in the CD11c(+) fraction at 1 and even 3 days after ablation. Despite the fact that the CD11c(+) DC only comprised a minor fraction of total LN cells, they accounted for up to 25% of total cell-associated activity present in the LN's (cryo ablation, not shown). Consistent with Figure 2B, uptake of ^111^In-KLH following radiofrequency ablation was significantly lower compared to cryo ablation. The counts in the CD11c(−) fractions were all below 100 (not shown).

These data obtained using marker model antigens thus strongly suggest that also tumour-debris created by ablation acts as an antigen depot and that released antigens preferentially accumulate in CD11c(+) DC in the draining LN. They also show that the method of destructing a tumour is highly relevant for the subsequent uptake dynamics of antigens.

### Accumulation of antigen-positive lymph node dendritic cell following ablation or conventional dendritic cell vaccination

**Figure 3:**
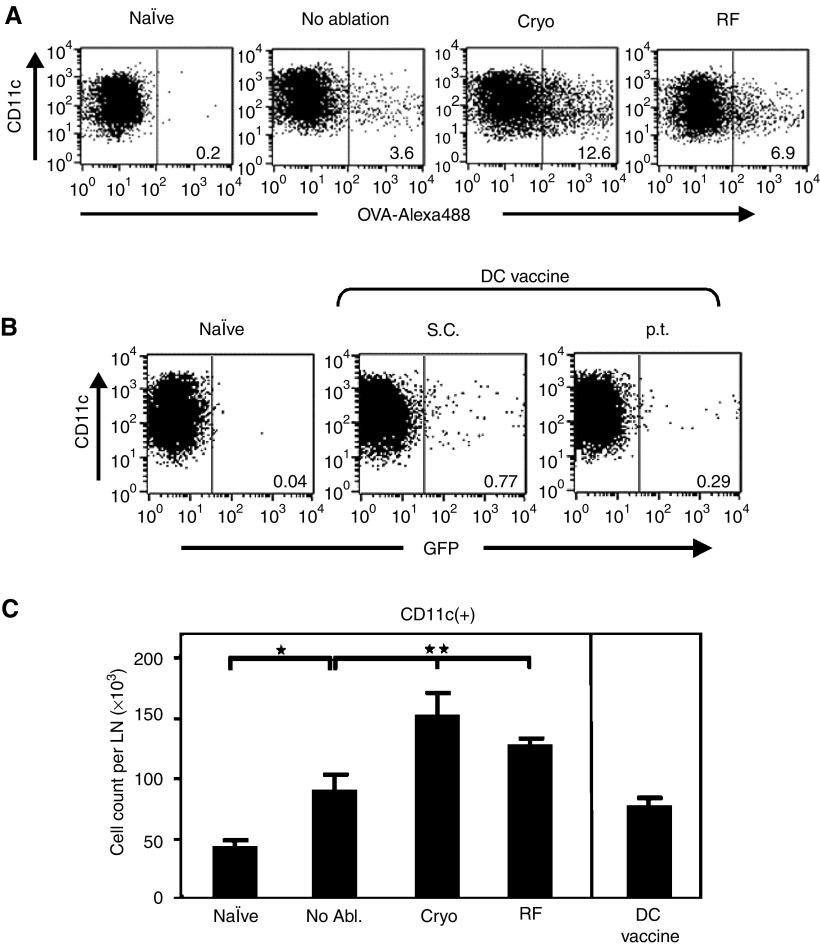
Increased numbers of antigen-positive DC in draining LN following ablation. (**A**) Fluorescence-activated cell sorting analysis of CD11c(+) DC isolated from pooled LN suspensions of naïve, tumour-bearing or tumour-ablated mice (*n*=6 per group). Mice received 20 *μ*g ovalbumin conjugated to Alexa-488 (OVA-Alexa488) i.t. just before the time point of ablation. Two days after the indicated treatments, CD11c(+) DC were isolated from draining LNs, stained for CD11c (clone HL3), gated and plotted for OVA-Alexa488 content. Values shown are percentages of OVA-Alexa488(+) cells within the CD11c(+) fraction. (**B**) Bone marrow dendritic cells were cultured from GFP-transgenic mice, loaded with peptides and matured *ex vivo* with LPS. Dendritic cells (1 × 10^6^ ) were injected p.t. in tumour-bearing mice or s.c. into naïve mice. Two days later CD11c(+) DC were isolated from pooled LN suspensions (*n*=6 per group), stained, gated and plotted for GFP. (**C**) Absolute CD11c(+) cell count per LN. Data are obtained from experiments described in A and B and presented as means with s.d. from three independent experiments. As controls for all experiments, naïve mice were used that did not receive any injection (naïve). ^*^=*P*<0.005 *vs* naïve, ^**^=*P*<0.01 both *vs* no ablation.

To compare the *in vivo* loading of DC by ablation with an externally loaded conventional DC vaccine, we analysed the numbers of antigen-positive LN DC after performing both techniques. In order to trace the antigen-experienced DC by flowcytometry, ovalbumin conjugated to the fluorophore Alexa-488 (OVA-Alexa) was injected i.t. before ablation and DC derived from GFP-transgenic mice were used as an *ex vivo* DC vaccine. These GFP DC were matured with LPS, loaded with the relevant OVA-K^b^ peptide (SIINFEKL) and 1 × 10^6^ DC were next injected s.c. or around an established (5–7 mm) B16-OVA tumour (p.t.). As shown in Figure 3A, over 12% of all draining LN CD11c(+) DC became OVA-Alexa(+) after cryo ablation, whereas after radiofrequency ablation 7% of DC acquired the antigen. These data are in line with the accumulation of antigens in DC observed with ^111^In-proteins (see also Figure 2). Consistent with other studies (Josien *et al*, 2000; De Vries *et al*, 2003; Eggert *et al*, 2003), only small numbers (<1%) of the *ex vivo* generated GFP DC reached the draining LN (Figure 3B). Moreover, plotting the *absolute* numbers of DC present in the draining LN further revealed a significant increase in total DC numbers after ablation, accompanied by an increased LN volume (Figure 3C and not shown). This implies that the absolute number of DC actually loaded with tumour antigen is even higher than can be concluded from the relative percentages of antigen-loaded DC shown in Figure 3A.

These data thus indicate that ablation is far more effective in obtaining marker antigen-loaded DC in the draining LN as compared to conventional DC vaccination.

### Ablation-dependent maturation of antigen-positive lymph node dendritic cell

**Figure 4:**
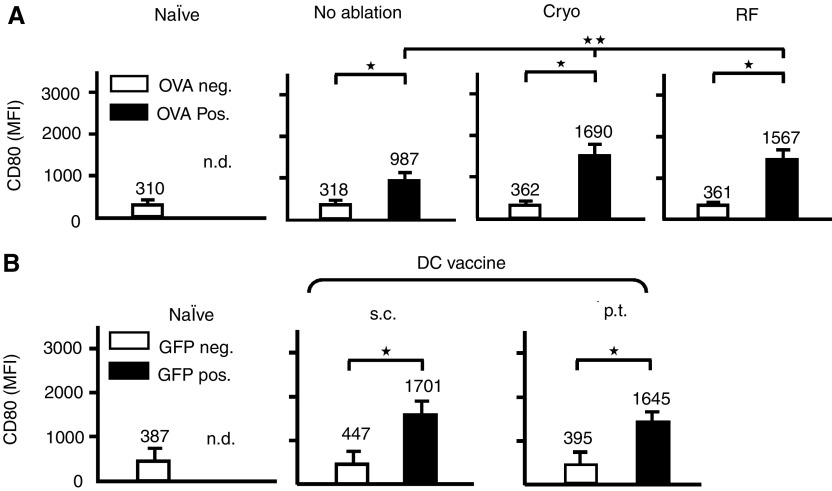
Ablation induces maturation of antigen-positive DC. OVA-Alexa488-positive or -negative CD11c(+) cells (**A**), or GFP-positive or negative CD11c(+) cells (**B**), obtained in the experiments shown in Figure 3, were analysed separately for expression of the maturation marker CD80. Values indicated are mean MFI's with s.d. from three independent experiments. ^*^=*P*<0.005, ^**^=*P*<0.05 both *vs* no ablation.

Next, we studied the maturation of DC in relation to antigen uptake. Therefore, OVA-Alexa(+) or OVA-Alexa(−) (in vaccination: GFP(+) or (−)) DC were analysed for expression of the DC maturation marker CD80. OVA-Alexa(+) DC showed a three-fold increase in CD80 expression relative to OVA-Alexa(−) DC in tumour-bearing and naïve mice (MFI's 987, 318 and 310, respectively) (Figure 4A). Moreover, CD80 expression further increased following cryo or radiofrequency ablation on OVA-Alexa(+) DC but had no effect on OVA-Alexa(−) DC (MFI 1690 *vs* 362 and 1567 *vs* 361, respectively). This indicates that DC that acquired OVA-Alexa, as an indicator of tumour-debris, preferentially upregulate CD80, whereas this effect is further enhanced by ablation. Importantly, the induction of maturation by radiofrequency ablation is equal to the induction by cryo ablation, even though less antigen uptake was observed. Analysis of the GFP-DC in the LN for CD80 expression, demonstrated that the exogenously loaded GFP(+) DC were significantly more mature than the resident endogenous GFP(−) DC (Figure 4B) The observed MFI's were comparable to those seen on antigen-positive DC after ablation and were similar after p.t. or s.c. injection (Figure 4B). Similar, but somewhat less profound results were obtained with CD86 expression (not shown).

The combined data thus not only indicate that ablation results in efficient DC loading but also that ablation-induced CD80 expression of the OVA-Alexa(+) DC in the LN equals the CD80 expression on the DC from the LPS-matured *ex vivo* DC vaccine.

### CTLA-4 blockade following ablation enhances systemic anti-tumour immunity

**Figure 5:**
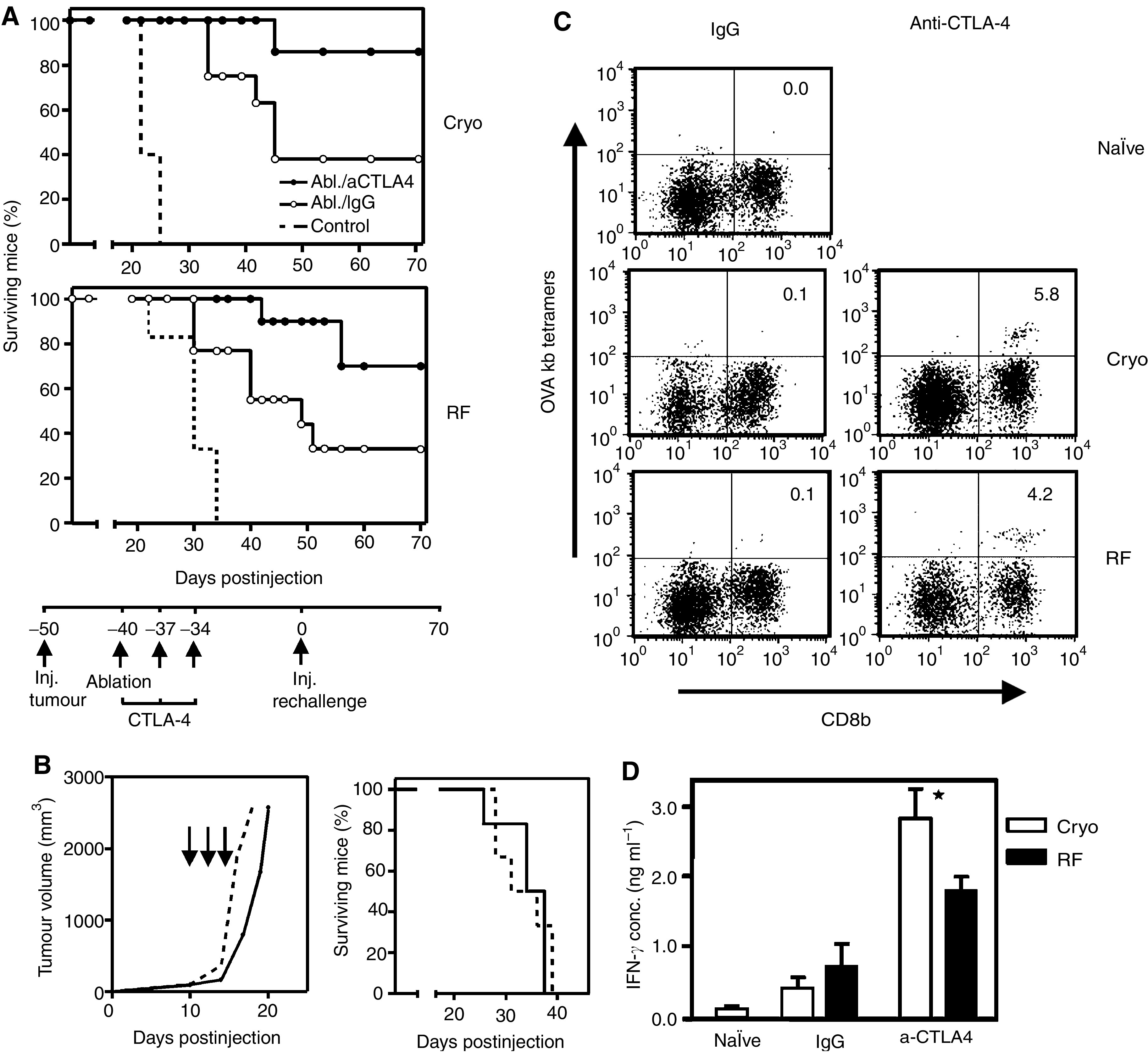
Anti-CTLA-4 improves therapeutic outcome of ablation. 0, 3 and 6 days after ablation of B16-OVA tumours, mice were injected i.p. with 200 *μ*g anti-CTLA-4 antibodies or control IgG. (**A**) Forty days following ablation of tumour-bearing mice receiving antibodies, a tumour re-challenge was performed as described before. Figures depict survival curves demonstrating growth reduction/protection after ablation plus CTLA-4 treatment. As a control, tumour growth was monitored by injection of the same tumour dose into naïve mice (dotted lines). *T*=0 corresponds to the time of injection of the tumour re-challenge. *P*<0.05 for CTLA-4 *vs* IgG in both cryo and RF figures. One out of three representative experiments is shown (*n*=5–11). (**B**) Control experiment showing that CTLA-4 treatment by itself is insufficient to eradicate the primary tumour or re-challenges. Mice with established B16OVA tumours (5–7 mm) were injected with 200 *μ*g anti-CTLA-4 antibodies at days 10, 13 or 16 after tumour inoculation (solid line, arrows) or PBS (dotted line). Next, tumour growth was monitored in time (left panel). CTLA-4 treatment or PBS given 40 days before a B16OVA challenge (15 × 10^3^ cells) did not affect survival of the mice (right panel). (**C**) At day 10 after ablation, a mix of LN and spleen cells was obtained from mice treated as indicated. T cells were harvested from spleen and LN and restimulated with irradiated, IFN-*γ*-treated B16-OVA cells and IL-2 for 10 days, followed by staining with OVA tetramers (K^b^) and anti-CD8b. Depicted numbers represent the percentages of tetramer-positive cells within the CD8b+ population. (**D**) T cells from the same bulk cultures were used for restimulation with B16OVA cells. Supernatant from these cultures was harvested 24 h later and analysed for IFN-*γ* content by standard ELISA methods. Shown are means with s.d. from triplicates, ^*^=*P*<0.05 *vs* IgG. Experiments shown in figures (**B**–**D**) were repeated twice with comparable results.

We previously showed that immune modulation by blockade of CTLA-4 signalling enhances anti-tumour immunity after radiofrequency ablation. We now studied whether CTLA-4 blockade could be also used in the cryo ablation model. Figure 5 shows that the weak anti-tumour response observed after cryo ablation alone indeed could be enhanced by combination with CTLA-4 blockade. Comparable results were obtained when radiofrequency ablation was applied (Figure 5A). Control experiments showed that CTLA-4 injection alone had no significant effect on primary tumours or re-challenges (Figure 5B, left and right panel, respectively). Analysis of the mice for OVA-specific T cells revealed that far more specific T cells were present 10 days after the combination treatment as compared to ablation alone (Figure 5C). To determine the activation status of these T cells, IFN-*γ* production was measured after activation with B16-OVA cells. Interestingly, T cells derived from mice treated with anti-CTLA-4 and ablation showed increased IFN-*γ* production upon recognition of antigen compared to the IgG treatment (Figure 5D). In correspondence with the re-challenge model, somewhat less IFN-*γ* producing, specific T cells were detected after radiofrequency ablation than after cryo ablation.

### Depletion of regulatory T cells before ablation enhances systemic anti-tumour immunity

**Figure 6:**
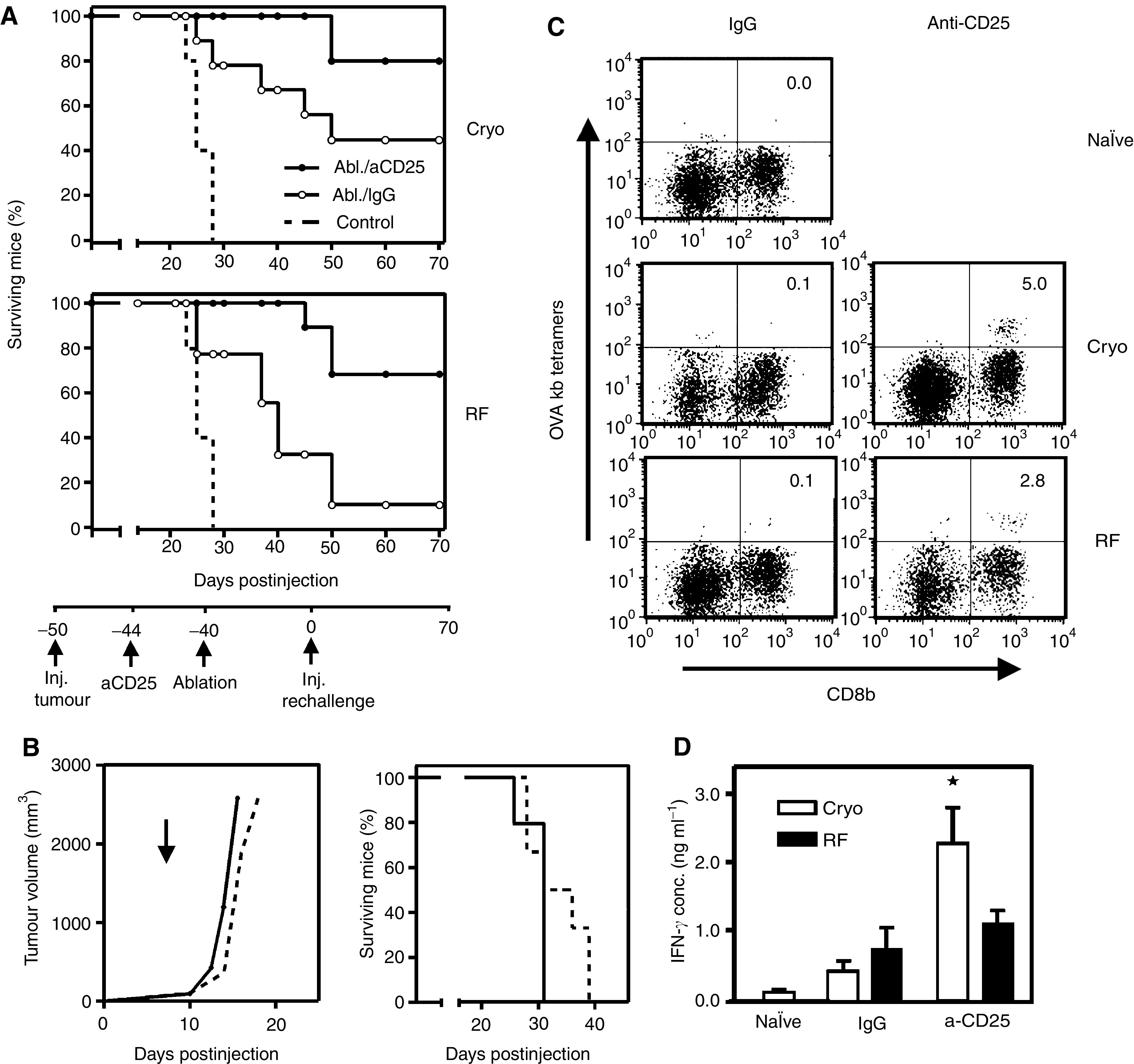
Depletion of regulatory T cells improves therapeutic outcome of ablation. Four days before ablation of B16-OVA tumours, mice were injected i.p. with 200 *μ*g anti-CD25 antibodies or control IgG. (**A**) Forty days following ablation of tumour-bearing mice receiving antibodies, a tumour re-challenge was performed as described before. Figures depict survival curves demonstrating growth reduction/protection after ablation plus Treg depletion. As a control, tumour growth was monitored by injection of the same tumour dose into naïve mice (dotted lines). *T*=0 corresponds to the time of injection of the tumour re-challenge. *P*<0.05 for aCD25 *vs* IgG in both cryo and RF figures. One out of three representative experiments is shown (*n*=5–9). (**B**) Control experiment showing that Treg depletion by itself is insufficient to eradicate the primary tumour or re-challenges. Mice with established B16OVA tumours (5–7 mm) were injected with 200 *μ*g anti-CD25 antibodies (solid line, arrow) or PBS (dotted line). Next, tumour growth was monitored in time (left panel). Treg depletion or PBS given 40 days before a B16OVA challenge (15 × 10^3^ cells) did not affect survival of the mice (right panel). (**C**) At day 10 after ablation, a mix of LN and spleen cells was obtained from mice treated as indicated. T cells were harvested from spleen and LN and restimulated with irradiated, IFN-*γ*-treated B16-OVA cells and IL-2 for 10 days, followed by staining with OVA tetramers (K^b^) and anti-CD8b. Depicted numbers represent the percentages of tetramer-positive cells within the CD8b+ population. (**D**) T cells from the same bulk cultures were used for restimulation with B16OVA cells. Supernatant from these cultures was harvested 24 h later and analysed for IFN-*γ* content by standard ELISA methods. Shown are means with s.d. from triplicates, ^*^=*P*<0.05 *vs* IgG. Experiments shown in figures (**B**–**D**), were repeated twice with comparable results.

Next, we investigated whether depletion of regulatory T cells before ablation was also able to enhance tumour immunity. Hereto, mice received anti-CD25 antibodies 4 days before ablation of their B16-OVA tumours. The anti-CD25 antibodies did not have any effect on the outgrowth of the B16-OVA tumours, yielding similar tumour-sizes at the day of ablation (see also Figure 6B). Figure 6A shows that Treg depletion enhances the initially weak anti-tumour responses after both ablative techniques. This effect is comparable to the effects seen with anti-CTLA-4 treatment (see also Figure 5). Treg depletion alone had no significant effect on primary tumours or re-challenges (Figure 6B). Tetramer analysis confirmed the presence of IFN-*γ* producing, tumour-specific T cells after the combination treatment, whereas after each individual treatment these cells were absent (Figure 6C and D). In correspondence with the re-challenge model, somewhat less specific T cells were detected after radiofrequency ablation then after cryo ablation.

Collectively, these data thus suggest that antigen uptake following ablation does only lead to enhanced numbers of activated tumour-specific T cells, when suppressive regulation is switched off. *In situ* tumour destruction plus immune activation leads to a more potent systemic anti-tumour response than either treatment modality alone. This treatment regimen allows for direct antigen loading of DC *in vivo* without delivery of defined tumour antigens as in conventional DC vaccination.

## DISCUSSION

*Ex vivo* generated mature DC have been shown to evoke tumour-specific responses in cancer patients (Figdor *et al*, 2004; Rosenberg *et al*, 2004). Dendritic cells vaccination is, however, time consuming and expensive, and in many cases the anti-tumour response falls short in strength to cure patients with established tumours. Herein, we report that tumour debris created by radiofrequency and cryo ablation comprises an effective antigen source for DC. Moreover, we show that tumour ablation could be efficiently combined with immune modulating approaches. This creates an effective ‘*in situ* DC-vaccine’ capable of inducing protection against lethal tumour re-challenges.

*In situ* tumour destruction with cryo, radiofrequency or laser ablation has received increasing attention as a treatment modality for focal cancer (Ruers *et al*, 2001; Ruers and Bleichrodt, 2002; Curley, 2003; Erce and Parks, 2003; Veth *et al*, 2005). However, little is known regarding the induction of immune responses after *in situ* tumour destruction or the fate of the generated tumour debris. In our experiments we applied a mouse B16 tumour model, in which *ex vivo* generated DC vaccines are mostly only effective in a *prophylactic* setting. Applying two types of ablation techniques and different types and labelled model antigens, we now demonstrated that antigen remaining *in situ* after tumour destruction creates an effective antigen depot for the induction of *therapeutic* anti-tumour immunity by DC. We applied two distinct exogenous antigens (OVA and KLH) and two different approaches (radioactive and fluorescent labelling) to monitor the fate of antigens. Although it remains difficult to exclude that these model antigens do not behave like tumour debris in all facets, based on our as well as published results it is reasonable to assume that they provide relevant information regarding the flow of tumour debris (Itano *et al*, 2003). We showed that the majority of antigens remained at the ablated site and that very little antigen spreading was observed except to the draining LN. Within the draining LN, a large percentage of DC acquired antigen as soon as 1 day and for at least 3 days following ablation. The *in vivo* loading of DC upon cryo ablation was significantly more efficient than with radiofrequency ablation (Figures 2 and 3). The exact nature of this difference remains to be elucidated but is likely related to the kind of antigens that are created by the ablation and/or to the endogenous signals that are produced upon tumour destruction.

Our results do not provide answers on *how* exactly DC acquire their antigens. It is, for instance, not known whether DC travel to the tumour and take up the antigens locally, or that the antigen floats to the LN via lymphatics, where LN resident DC engulf this material. According to a recent study demonstrating LN-DC that accumulated antigen deposited in s.c. tissue (Itano *et al*, 2003), both options might be occurring at the same time. The authors showed that antigen was first detected in LN-residing DC, followed by a second wave of antigen-positive DC that migrated from the periphery into the LN. Both waves were required for efficient immune response induction and were dependent on the presence of the challenge site. Our finding that both at day 1 and day 3 after ablation antigen-loaded DC could be discerned from the LN suggests that similar dynamics take place in our model. In this context, it is interesting to note that antigen loading in tumour bearing control mice seemed to decline in these 3 days (see also Figure 2D). Furthermore, we observed that antigens from the tumour depot preferentially accumulate in DC, but not in B-cells or macrophages (not shown). The basis for the observed antigen accumulation in DC remains to be studied in more detail, but is likely related to their strategic location within the LN and their ability to retain antigens within their endocytotic compartment, whereas macrophages rapidly degrade antigens in their lysosomes.

The primary goal of conventional DC-based vaccination is to obtain tumour antigen-loaded DC in the draining LNs that are properly activated so that they do initiate immune responses. Although the nature of the antigens brought to the LN by DC loaded via ablation *vs* antigen-positive DC from a conventional DC vaccine is difficult to compare, we could demonstrate that far more antigen-loaded DC were present in the draining LNs when radiofrequency or cryo ablation was performed (Figure 3). Moreover, both ablation procedures were able to significantly increase the absolute number of DC per LN, whereas vaccination was less able to do so (Figure 3C). Analysis of the maturation state of antigen-loaded and -unloaded DC in naïve, tumour-bearing and tumour-ablated mice revealed two interesting phenomena. First, DC that contained antigen expressed significantly higher levels of co-stimulatory molecules than antigen negative DC. These data are in line with *in vitro* data indicating that antigen uptake can affect DC activation (Randolph *et al*, 1998). Second, ablation resulted in a significant further increase in co-stimulatory molecule expression on antigen positive, but not antigen-negative DC. We note that neither antibodies present after positive MACS sorting, nor TLR ligands often present in OVA batches (Pasare and Medzhitov, 2004) did bias the CD80 staining on DC, as DC purified by negative selection showed similar results and OVA-Alexa did not mature DC *in vitro* (not shown). The exact nature of these ablation-dependent signals need further clarification, but may well represent cytokines or other endogenous mediators released after ablation (de Jong *et al*, 2001; Huang *et al*, 2002; Pasare and Medzhitov, 2004; Sporri and Reis e Sousa, 2005). It has, for instance, been shown that heat shock proteins and uric acid are present in cell debris, which both influence DC and other parts of the immune system (Somersan *et al*, 2001; Shi *et al*, 2003). Importantly, the observed ablation-induced increase in the number and maturation state of antigen-loaded DC is apparently not sufficient to induce complete tumour protection of mice.

Combination of ablation with *in vivo* immune modulation by either blockade of CTLA-4 signalling or depletion of regulatory T cells was shown to have beneficial effects in our re-challenge model. In both cases it provided a further delay in tumour growth compared to ablation alone. Importantly, only when CTLA-4 blockade or regulatory T-cell depletion were performed together with ablation, significant amounts of active OVA-specific T cells could be observed. It will be interesting to investigate the nature of the signals resulting in DC maturation and subsequent T-cell expansion, and the possible effects of CTLA-4 blockade or Treg depletion on this. Moreover, possible future research can aim on combination of CTLA-4 blockade and Treg depletion. It was demonstrated in a murine tumour model that this combination had a striking synergistic effect on tumour immunity (Sutmuller *et al*, 2001).

Collectively, our data show that *in vivo* tumour destruction in combination with systemic immune modulation creates a unique and potent, ‘*in situ* DC-vaccine’. Although radiofrequency ablation seems to be somewhat less efficient in loading DC compared to cryo ablation, both techniques can be efficiently combined with immune modulation. The fact that both ablative treatments as well as both the immune interventions are currently applied in cancer patients, makes this promising ‘*in vivo* DC vaccine’ readily applicable in clinical settings.
